# Recent advances in connexin gap junction biology

**DOI:** 10.12703/r/11-14

**Published:** 2022-05-27

**Authors:** Paul D Lampe, Dale W Laird

**Affiliations:** 1 Translational Research Program, Fred Hutchinson Cancer Center, Seattle, WA, USA; 2 Department of Anatomy and Cell Biology and Department of Physiology and Pharmacology, University of Western Ontario, London, ON, Canada

**Keywords:** connexin, gap junctions, channel, hemichannel

## Abstract

Connexins are assembled into dodecamer intercellular channels, a collection of which is termed a gap junction, and their canonical function allowing direct exchange of ions and metabolites has been unequivocally established. When initially assembled into undocked cell surface connexin hemichannels, healthy cells may also engage in cell signaling via a regulated small-molecule release. Recent advances in the field have led to an expanded view of the functional roles of intercellular channels and hemichannels in both physiology and pathology. As more of the 21-member human connexin family is intensely interrogated, mounting evidence points to the biological uniqueness of each member, and no longer can we confidently refer to all connexins engaging in the same cellular processes. Innovations in high-resolution cryo-electron microscopy have revealed important insights into the structure of functionally important domains of both hemichannels and channels. These and other studies have established a foundation of knowledge that should allow inhibitory smart drug design for situations where enhanced intercellular or hemichannel activity is at the root of a connexin-linked disease. Assessment of the connexin interactome, which varies widely for each connexin subtype, continues to provide regulatory insights into the assembly and function of connexins that exhibit a short half-life. As the most intensely studied, Cx43 is found in about 50% of all human cell types and is extensively regulated by multiple inhibitory and enhancing phosphorylation events that have direct implications on tissue function and outcomes of disease, including cancer. Here, we briefly discuss these advances and give our thoughts on where the field is headed.

## Introduction

**Figure 1.**
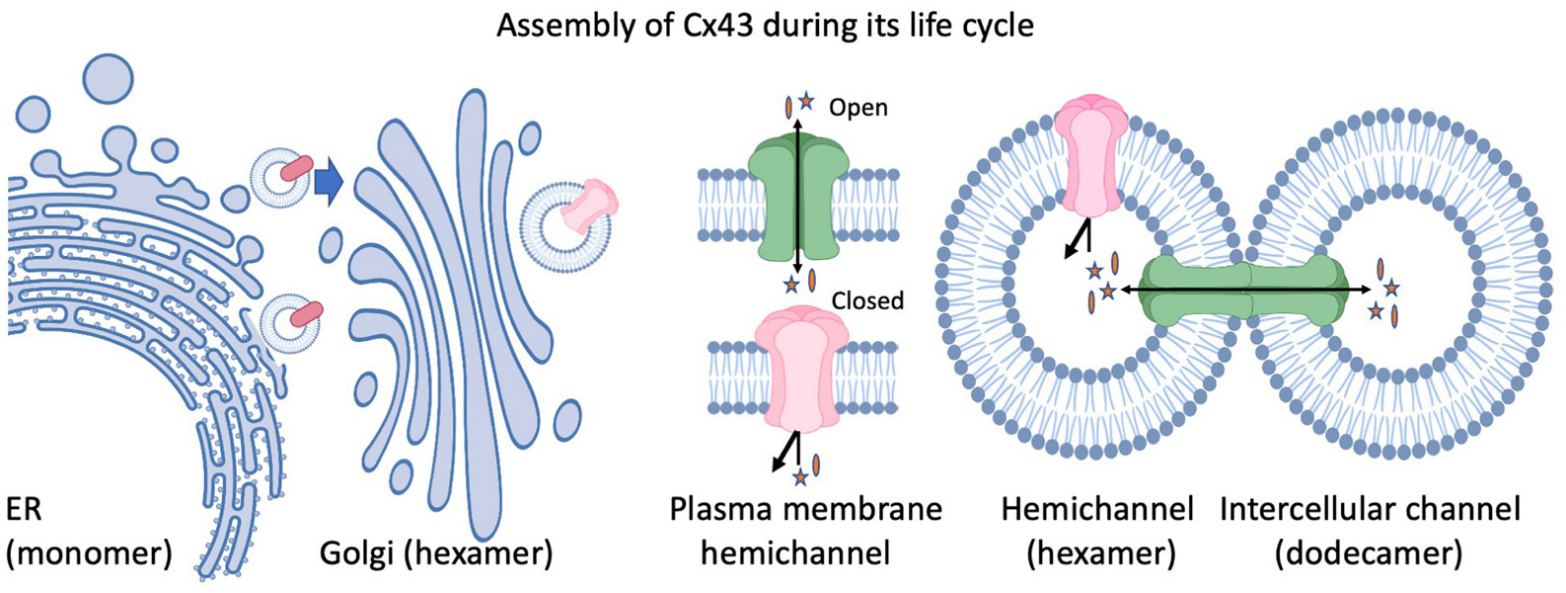
Assembly of Cx43 during its life cycle. Cx43 is produced in the endoplasmic reticulum (ER) and is exported to the Golgi apparatus as a monomer. In the Golgi, Cx43 oligomerizes into hexamers and is exported to the plasma membrane, where it can exist as a closed (red channel) or open (green) hemichannel that can allow the regulated passage of specific small molecules (denoted by stars and ovals). Hemichannels from adjoining cells dock and form a head-to-head dodecameric structure (gap junction channel) to permit the regulated intercellular passage of small molecules and metabolites.

The most well-known canonical functions of gap junctions are to permit and regulate the intercellular exchange of hundreds of small cellular metabolome constituents^1,2^. Gap junctions play both structural and intercellular communication roles to help regulate many cell processes, including cell migration, cell proliferation, embryonic development, differentiation, wound repair, and the coordinated contraction of heart and smooth muscle^2^. There is unequivocal evidence that, in humans, the channel-lining proteins of gap junctions are composed of proteins from the 21-member connexin gene family^3^ (Figure 1). Genetic linkage analyses have associated 11 of these connexins to at least 30 human diseases with broad phenotypes, including deafness, syndactyly, skin diseases, neuropathies, lymphedema, cataracts, and developmental defects, emphasizing the fact that connexins are expressed in a tissue-specific manner^4^. However, not only are connexins linked to disease through gene mutations, but their role in cancer continues to be a focal point of research as the community continues to assess the potential value of prioritizing at least some connexins as therapeutic targets for specific tumor types or in cancer stem cells^5–8^. Still, other studies have identified the value of transiently downregulating connexin expression or function in diseases of the eye and in diabetic wounds^2,9,10^. There remains no doubt that connexins play critical roles in healthy cells as well as in pathophysiology. New advances in the areas of gap junction structure and regulation continue to expand the breadth of functions linked to connexins in cell biology.

## Revisiting the canonical function of connexins

Interrogation by X-ray diffraction, freeze-fracture electron microscopy, nuclear magnetic resonance, and atomic force microscopy, together with sophisticated image analysis, revealed that the dodecameric arrangement of connexins was needed to form a complete intercellular channel^11–14^. Direct confirmation of the connexin arrangement and molecular details of the pore-lining domains were lacking until 2009 when Maeda and colleagues solved the structure of the Cx26 gap junction channel at a resolution of 3.5 Å^15^. New details, such as the identification of positively charged residues at the cytoplasmic entrance to the channel, began to emerge from these high-resolution structures^15^. More recently, it was shown that, when calcium bound to Cx26, the electrostatic barrier changed to prevent the passage of channel permeants, a process previously proposed to be related to a wholesale movement of connexin subunits^16^. The emergence of single-particle image analysis and cryo-electron microscopy (cryo-EM) accelerated the structural revolution as high-resolution images of native lens gap junctions composed of Cx46 and Cx50 were obtained^17^. Here, the authors definitively proved the transmembrane domain arrangements and found open conformational states that were distinct from Cx26^17^, possibly revealing differences between alpha and beta connexin family members. This same team further resolved the structure of Cx46/50 channels in a dynamic aqueous-lipid context at near atomic resolution (1.9 Å), where the connexin proteins were found to stabilize the local lipid microenvironment^18^. This level of molecular detail is now sufficiently informative to serve as a model for rational drug design for the possible treatment of any number of inherited connexin-linked diseases^4,8^ and the countless pathologies linked to connexin regulation in diseased tissue and cancer^19^.

While connexins form intercellular channels that permit the direct passage of metabolomes, the 1991 discovery that macrophages could release ATP, via what the authors termed “half-gap junctions” or what today we would call hemichannels, expanded our view of connexin function^20^. In the years that followed, the community had to consider that connexins may, in fact, have a second canonical function in forming cell surface channels capable of releasing (or, on occasion, internalizing) metabolites or a variety of signaling molecules or both. While this core cellular function has molecular rivals, most notably pannexin channels^21,22^, there was no doubt that connexin hemichannel functions needed to be considered in both physiological and pathological contexts. The question continues to arise as to where and when connexin hemichannels play a role in normal healthy physiology and where and when aberrant hemichannel activity would lead to cell death or disease. No fewer than 1500 peer-reviewed papers have featured the existence and functional role of connexin hemichannels in virtually every organ of the human body. These studies range from Cx43 hemichannels engaging in a ventricular arrhythmogenic mechanism within microdomains of cardiac intercalated discs^23^ to Cx43 hemichannels in astrocytes serving as a therapeutic target in spinal cord injury^24^ and to Cx43 hemichannels in osteocytes playing a critical role in mechanical load-induced bone formation^25^. Intuitively, connexin intercellular channels tend to favor an open state, as supported by recent structural experimental evidence, but our view is that hemichannels must have a resting closed state under homeostatic conditions in order to protect the integrity of the cytosol^17,18,26–30^.

However, evidence continues to mount that hemichannels play a more universal and canonical functional role in non-diseased tissues. As an example, considerable evidence suggests that Cx31.3 may not even have the capacity to form traditional gap junction intercellular channels, raising the notion that their main role in cells may be to act as functional hemichannels^31^. Likewise, the evidence for functional Cx46 and Cx50 hemichannels in highly specialized lens tissue in the presence and absence of disease is compelling^32,33^. Still, other less well-studied connexins like Cx62 may have dual roles in platelets, functioning as both hemichannels and intercellular channels^34^. One must also consider that connexin hemichannels may only partially open, permitting the smallest of channel permeants to pass. Insight into this notion was provided when cryo-EM was used to solve the hemichannel structure of a connexin that appears unable to form functional gap junction channels, Cx31.3^27,31^. Resolution analysis that exceeded 2.6 Å revealed that Cx31.3 hemichannels adapt to a partially closed state that would allow the passage of chloride ions through the 8 Å pore while preventing the passage of other cell metabolites^27^. Cryo-EM was also used to examine the open conformation of Cx26-N176Y mutant hemichannels within dynamic lipid bilayer nanodiscs^28^. Here, the alpha-helical structures found within the mutant Cx26 hemichannels were found to be the same as reported for Cx26 intercellular channels while the flexibility identified within the extracellular loops would probably serve to facilitate hemichannel docking in cases where complete gap junction channels are formed^28^. However, likely owing to their intrinsic disorder, we still know little about the structure of intracellular loop and C-terminal tail regions. Thus, there is no doubt that further high-resolution analysis of different connexin hemichannels and intercellular channels will continue to inform on their open and closed states.

To our minds, there remains little doubt that aberrant connexin hemichannel function is at the root of several inherited connexin-linked diseases and cases of tissue injury as well as in chronic and acute diseases. Many missense and truncating connexin-gene mutations lead to hyperactive or leaky hemichannels that appear especially prevalent in connexin-linked skin diseases^4,35,36^. Mutations in this class often lead to the loss of cell integrity and cell death, but other mechanisms of action need to be considered^4,35,36^. Blocking connexin hemichannels as a means of regulating the inflammatory response has given credence to the notion that this may be an effective strategy to treat chronic inflammatory eye diseases and eye injuries^9^. In fact, hyperactive or leaky hemichannels in disease or injury are somewhat ideal targets when considering commercialization and deployment of connexin hemichannel blockers^2,8,37^. Preferably, such hemichannel blockers should be specific to the aberrant hemichannel in question, a consideration that aligns with smart small-molecule design modeled from high-resolution connexin hemichannel structures, peptide mimetics that take into account both structure and domain flexibilities, and high-avidity antibodies. As an example, an antagonist antibody was shown to have efficacy in blocking leaky mutant Cx30 hemichannels in the treatment of Clouston syndrome^38^. Similarly, the hemichannel blocker flufenamic acid was found to inhibit aberrant Cx26-G45E hemichannel function**,** reducing the symptoms of keratitis ichthyosis deafness found in mutant mice^39^. As noted by others, several connexin-based therapeutics that have entered clinical trials need to consider the specificity of the connexin blocking agent and whether the pathological features of hemichannels can be selectively and effectively targeted^40^.

Finally, while connexins are foundational molecules needed to assemble both hemichannels and gap junction channels, we would be remiss if we did not draw attention to the connexin interactome. The connexin interactome is highly dependent on the connexin family member as some interactomes are small (e.g., Cx26), while others are in excess of 50 proteins (e.g., Cx43)^41^. In the case of Cx43, the interactome includes proteins involved in protein trafficking, connexin turnover, connexin assembly, connexin post-translational modification, scaffolding, and other functions that have been extensively reviewed elsewhere^41,42^. The gap junction scaffold is also functionally important for other connexins, as has been shown for Cx30 in the cochlea, where ephrin-B2 interacts at the periphery of the gap junction regulating gap junction turnover^43^ in a manner perhaps analogous to ZO-1 interaction with Cx43^44^. We believe the scaffold function of gap junctions will be found to play critical regulatory roles as more research is performed on other connexins, and we look forward to new revelations.

## Regulation of gap junction channels by phosphorylation

**Figure 2.**
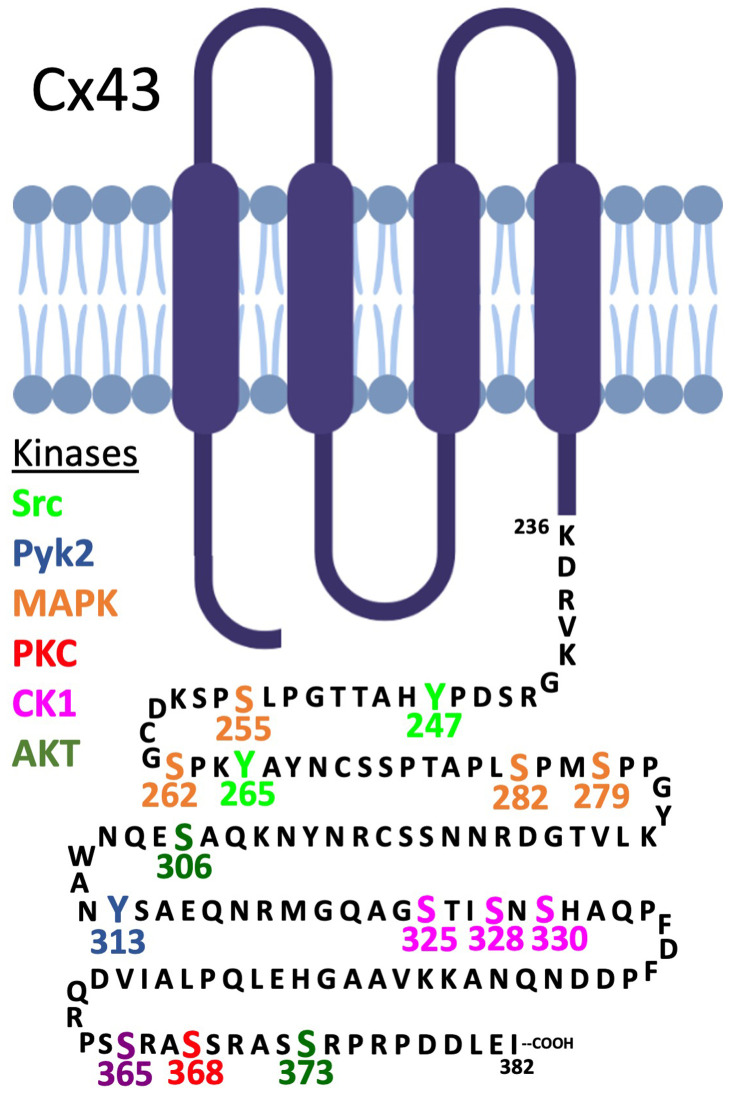
Map of the C-terminal tail of Cx43 with phosphorylation sites having known regulatory functions indicated. The specified protein kinases and the Cx43 residues they are known to be phosphorylated by are denoted by distinct colors. The site at S365 (purple) is phosphorylated by an unknown kinase that is influenced by protein kinase A activity (i.e., the effect is likely indirect). CAMKII has also been reported to phosphorylate several additional residues *in vitro*.

Our current understanding of the regulation of gap junctions is heavily biased toward Cx43. Cx43 is expressed in most cell lines even if they originated from cells that do not typically express Cx43 (e.g., hepatocytes express Cx32 and Cx26, but cell lines derived from them typically express only Cx43). Furthermore, our analysis of the literature suggests that Cx43 is natively expressed in nearly half of the more than 200 cell types found in the human body. Many Cx43 post-translational modifications, including ubiquitination, acylation, hydroxylation, carboxylation, methylation, sumoylation, and nitrosylation, have been reported, but we know the most about the functional consequences of Cx43 phosphorylation^45^. It is now clear that more than eight protein kinases phosphorylate Cx43 at most of the 21 serine residues and at least three of the six tyrosine residues found within the cytoplasmic exposed carboxy terminus (Figure 2). We also know that phosphorylation regulates the transport of Cx43 to the plasma membrane and its assembly into gap junctions and channel gating^46^, but more recent studies have also linked Cx43 phosphorylation events to gap junction stability and turnover^46–50^. At the level of tissue and organ physiology, recent evidence indicates that the Cx43 phosphorylation status is intimately linked to cardiac disorders, cardioprotection, ameloblast differentiation, oocyte maturation, angiotensin II-induced renal damage, B-lymphocyte spreading, epidermal wound repair, and autophagy^51–57^. Evidence for the essential role of some of these Cx43 phosphorylation events has been obtained via the use of genetically modified mice where known phosphorylation sites were modified to unphosphorylatable residues (e.g., serine to alanine) or to mimic a constitutively phosphorylated residue (e.g., serine to aspartate). Via this approach, casein kinase 1 phosphorylation of Cx43 was found to regulate several critical physiological events, including (a) proper cardiac beat rhythm^58^ and response to ischemia^55,56^, (b) efficient epidermal wound healing^57^, and (c) the effects of stromal fibroblasts on promoting pancreas cancer progression^59^, while (d) MAPK phosphorylation was shown to regulate neuroprotection during stroke^60^.

Our knowledge of phosphorylation of connexins other than Cx43 and the resulting physiological effects is in general less well-defined. There are reports that Cx31, Cx32, Cx36, Cx37, Cx40, Cx43, Cx46, Cx47, and Cx50 are phosphorylated^45^, but in some cases the specific residue or direct linkage of the phosphorylation event to an effect on connexin-linked physiology is lacking. We conclude that many of the alpha subfamily of connexins that have been studied have shown at least some evidence of regulation by phosphorylation. However, some connexins, particularly those of the beta subfamily that have short C-terminal regions, have not been shown to be specifically regulated by phosphorylation (e.g., Cx26, likely Cx23), although caution should be exercised as the effects of phosphorylation might be apparent only under very specific conditions. In any case, we believe new interest in the less common connexins that are expressed in diverse tissues will more clearly delineate the roles connexin phosphorylation plays in gap junction and hemichannel regulation.

## Looking toward the future of connexin and gap junction research

After an exhaustive review of over 180 peer-reviewed papers related to endogenous connexin expression, we conclude that the 21-member connexin family can be mapped to over 110 distinct cell types found within all 12 human body systems. On one end of this spectrum, it is unclear what human cells express Cx23, while at the other end, Cx43 has been convincingly shown to be expressed in 92 cell types, reflecting its dominance as the most widely distributed human connexin. After decades of intense investigation, the gap junction community not surprisingly is best equipped to describe where and when Cx43 functions regulate cell and tissue physiology. That said, the functional roles of lesser studied connexins (e.g., Cx25, Cx59, and Cx62) remain poorly understood, a situation made more difficult by the lack of reliable antibodies to specifically detect their expression *in situ*. While connexin family members share considerable sequence homology, it is now clear that connexin members are remarkably diverse with unique structures, regulatory motifs, post-translational modifications, interactomes, cell expression profiles, and subcellular distributions that all contribute to diverse intercellular channel and hemichannel functions in cells and tissues.

New advances in understanding hemichannel functions, intercellular channel and hemichannel structures, genetic linkages to disease, and the connexin interactome all point to critical and canonical roles for connexin hemichannel and channel functions in both disease and normal physiology. Some new areas of intensive study include the role of hemichannels in cardiac conduction^23,61^, connexin expression at non-canonical sites such as exosomes^62^ and mitochondrial outer membranes^63^, as well as the role that connexins play in cancer invasion, metastasis, and resistance to treatment^64^. There remains no doubt that connexins regulate a variety of cellular functions in almost every human organ. However, the challenge remains to sort out the tissue-context roles played by hemichannels/channels, a task made more complicated by our relatively rudimentary knowledge of what metabolites pass through each channel subtype *in situ* and the fact that connexin subtypes can intermix to form heteromeric and heterotypic channels. As more and more connexins get linked to diseases, a broad spectrum of organ-specific investigators from around the world are being attracted to the gap junction field in search of new therapeutic targets. We wholeheartedly welcome these new investigators to the field and believe they will pave the way to a better understanding of the functional importance of connexin-based cellular communication in all tissues.
